# CELEBRIMBOR: core and accessory genes from metagenomes

**DOI:** 10.1093/bioinformatics/btae542

**Published:** 2024-09-19

**Authors:** Joel Hellewell, Samuel T Horsfield, Johanna von Wachsmann, Tatiana A Gurbich, Robert D Finn, Zamin Iqbal, Leah W Roberts, John A Lees

**Affiliations:** European Bioinformatics Institute, European Molecular Biology Laboratory, Wellcome Genome Campus, Hinxton, Cambridge CB10 1SD, United Kingdom; European Bioinformatics Institute, European Molecular Biology Laboratory, Wellcome Genome Campus, Hinxton, Cambridge CB10 1SD, United Kingdom; European Bioinformatics Institute, European Molecular Biology Laboratory, Wellcome Genome Campus, Hinxton, Cambridge CB10 1SD, United Kingdom; European Bioinformatics Institute, European Molecular Biology Laboratory, Wellcome Genome Campus, Hinxton, Cambridge CB10 1SD, United Kingdom; European Bioinformatics Institute, European Molecular Biology Laboratory, Wellcome Genome Campus, Hinxton, Cambridge CB10 1SD, United Kingdom; European Bioinformatics Institute, European Molecular Biology Laboratory, Wellcome Genome Campus, Hinxton, Cambridge CB10 1SD, United Kingdom; Milner Centre for Evolution, University of Bath, Bath BA2 7AZ, United Kingdom; European Bioinformatics Institute, European Molecular Biology Laboratory, Wellcome Genome Campus, Hinxton, Cambridge CB10 1SD, United Kingdom; Centre for Immunology and Infection Control, Queensland University of Technology, Brisbane, QLD 4000, Australia; European Bioinformatics Institute, European Molecular Biology Laboratory, Wellcome Genome Campus, Hinxton, Cambridge CB10 1SD, United Kingdom

## Abstract

**Motivation:**

Metagenome-Assembled Genomes (MAGs) or Single-cell Amplified Genomes (SAGs) are often incomplete, with sequences missing due to errors in assembly or low coverage. This presents a particular challenge for the identification of true gene frequencies within a microbial population, as core genes missing in only a few assemblies will be mischaracterized by current pangenome approaches.

**Results:**

Here, we present CELEBRIMBOR, a Snakemake pangenome analysis pipeline which uses a measure of genome completeness to automatically adjust the frequency threshold at which core genes are identified, enabling accurate core gene identification in MAGs and SAGs.

**Availability and implementation:**

CELEBRIMBOR is published under open source Apache 2.0 licence at https://github.com/bacpop/CELEBRIMBOR and is available as a Docker container from this repository. Supplementary material is available in the online version of the article.

## 1 Introduction

**Figure 1.**
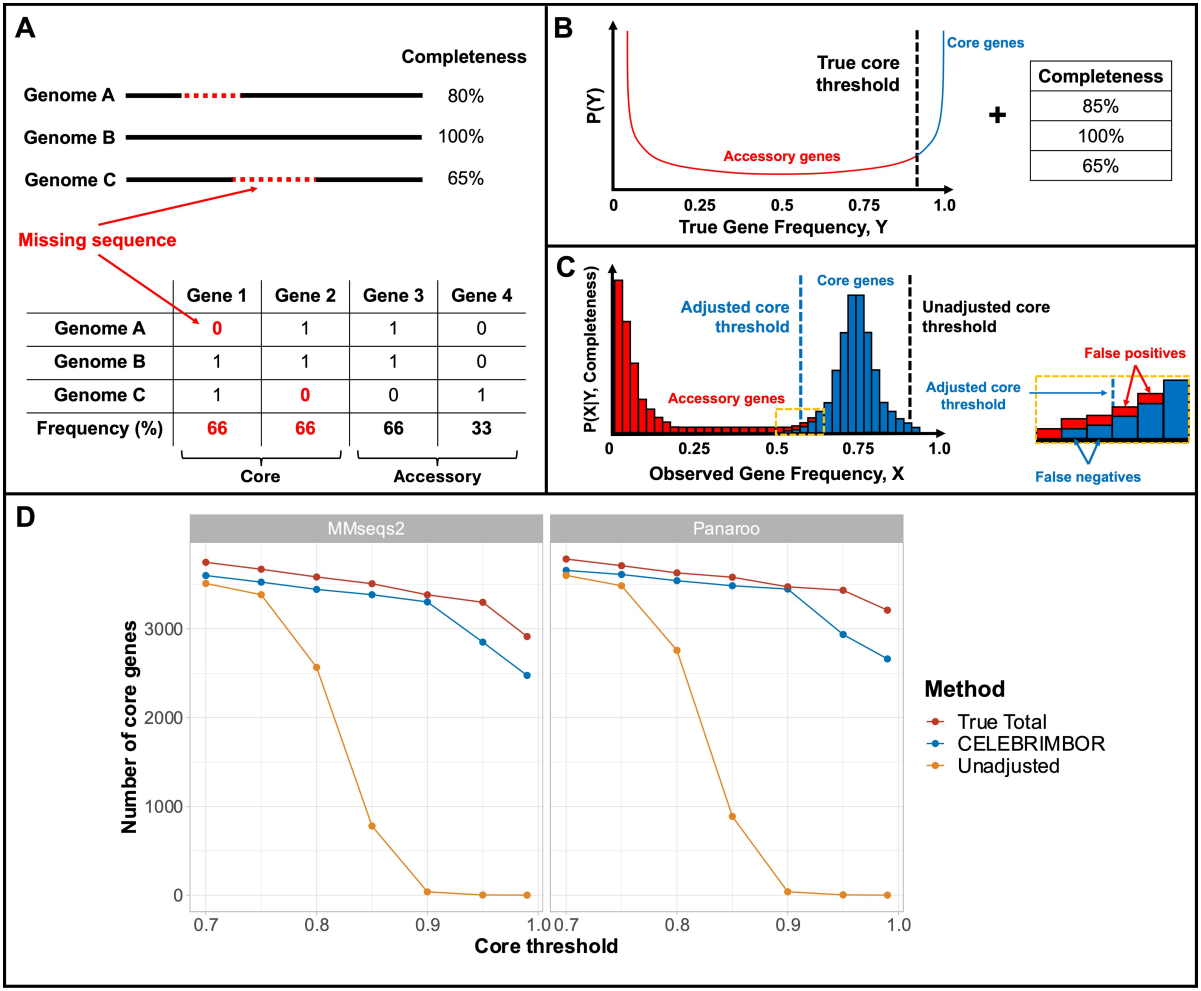
Overview of CELEBRIMBOR method and performance on simulated data. (A) Metagenome-assembled genomes (MAGs) and Single-cell Amplified Genomes (SAGs) are often incomplete, with missing sequences from inaccurate assemblies resulting in repeated absence of gene predictions in gene presence/absence matrices. As more genomes are included in pangenome analyses, the probability of a true core gene being missed in a MAG increases, meaning no core genes will be observed in a sufficiently large dataset. (B) CELEBRIMBOR uses a prior distribution of true gene frequencies, Y, given by a Beta, and completeness for each genome to simulate observed gene frequencies. The ‘true core threshold’ describes the frequency above which a gene is considered core, or below which it is considered accessory. (C) Observed gene frequencies describe the number of times a gene is observed in a set of incomplete assemblies, X, given a true frequency Y and genome completeness. CELEBRIMBOR adjusts the core genome boundary to capture true core genes with a degree of error specified by the user. The dashed box is a zoomed representation of the presence of errors around the adjusted core threshold; false negatives are true core genes that are observed at frequencies lower than the threshold, whilst false positives are accessory genes that are observed at frequencies higher than the adjusted threshold. The number of false negatives and positives can be controlled by adjusting the false negative rate (Supplementary Fig. S2). (D) Comparison of the number of core genes identified with varying core threshold and 5% false negative rate using a simulated *E.coli* dataset, where sequences were randomly removed from assemblies. Genes with a frequency at or above the core threshold were identified as core genes. ‘True Total’ describes the number of core genes identified prior to sequence removal. ‘CELEBRIMBOR’ and ‘Unadjusted’ refer to the CELEBRIMBOR-adjusted and unadjusted estimates of the number of core genes, respectively. Genes were clustered using MMseqs2 and Panaroo (strict mode).

Metagenome-assembled genomes (MAGs) and Single-cell Amplified Genomes (SAGs), generated from sequencing of complex microbial mixtures, make up a large proportion of publicly available bacterial genomes, with their abundance driven by bioinformatic pipelines designed specifically to reconstruct and assess the quality of MAGs and SAGs (Kieser *et al.* 2020, Tadrent *et al.* 2022, Gurbich *et al.* 2023, Schmidt *et al.* 2024). A key step in the analysis of a bacterial genome collection, including for MAGs and SAGs, is the identification of functionally identical genes, or orthologs, that are present in nearly all genomes for that species, known as ‘core’ genes, and differentiating them from ‘accessory’ genes present at lower frequencies (Baumdicker *et al.* 2012, Parks *et al.* 2018, Zhou *et al.* 2020, Tonkin-Hill *et al.* 2023). However, due to both uneven sequence coverage across genomes and assembly errors, MAGs and SAGs are often incomplete, with regions of a genome missing from the assembly (Fig. 1A). This results in fewer observations of genes and therefore systematic underestimation of core genome size (Li and Yin 2022).

Existing approaches that account for incomplete genomes when estimating core genome size use binomial or multinomial models which adjust a core frequency threshold; the frequency above which a gene is assigned to the core genome (Snipen *et al.* 2009, van Tonder *et al.* 2014). Such approaches rely solely on gene frequency estimates, and so are sensitive to errors in gene prediction and clustering (Tonkin-Hill *et al.* 2023). PPanGGOLiN (Gautreau *et al.* 2020) uses gene synteny in addition to a multinomial model of gene frequency to adjust multiple frequency thresholds, including that of the core genome. However, PPanGGOLiN was not designed exclusively for analysis of MAGs or SAGs, where systematic assembly errors may lead to a lack of contiguity, reducing identifiable synteny and therefore negatively impacting threshold adjustment. mOTUpan (Buck *et al.* 2022) uses a Bayesian approach which does not rely on synteny; genome completeness, an estimate of how much of a genome is represented by a given assembly based on core gene presence (Parks *et al.* 2015), is iteratively updated until convergence using a probabilistic model of gene frequencies.

In this work, we propose an alternative method for core frequency threshold adjustment using genome completeness, CELEBRIMBOR (Core ELEment Bias Removal In Metagenome Binned ORthologs, pronounced ‘Kelebrimbor’). CELEBRIMBOR uses genome completeness, jointly with gene frequencies, to adjust the core frequency threshold in a single step by modelling the number of gene observations with a true frequency using a Poisson binomial distribution.

## 2 Materials and methods

### 2.1 CELEBRIMBOR workflow

CELEBRIMBOR is a Snakemake workflow which conducts full pangenome analysis and core threshold adjustment. Genes are first predicted using Bakta (Schwengers *et al.* 2021) and then clustered to generate a gene presence/absence matrix using either MMseqs2 (Steinegger and Söding 2017), or Panaroo (Tonkin-Hill *et al.* 2020), depending on user preference. MMSeqs2 is run with minimum 90% sequence identity and 80% reciprocal sequence coverage between two sequences for clustering by default, whilst Panaroo can be run with one of three predefined clustering settings: strict, moderate, or sensitive (Tonkin-Hill *et al.* 2020). Genome completeness is calculated using conserved single-copy marker genes with CheckM (Parks *et al.* 2015). The presence/absence matrix and completeness are input to the core genome threshold calculation (cgt, https://github.com/bacpop/cgt), which implements the probabilistic threshold adjustment method described below.

### 2.2 Core threshold adjustment

Customarily, genes are identified as core genes if they appear in 95% or more of genome samples (99% is another common choice) (Tonkin-Hill *et al.* 2020). Due to incomplete genomes, genes with a true frequency of 95% or more can be observed in <95% of samples. We propose the following probabilistic model to resolve this issue.

Over N genome samples in a genome dataset, the total number of times that a gene is observed is the sum of successes in N independently distributed Bernoulli trials that each have the probability of success ${\theta c}_{1},\;\ldots ,\;\theta c_{N}$ where $c_{i}$ is the completeness score of the *i*th genome sample and θ is the true gene frequency. The number of observations of a gene, X, is distributed according to a Poisson Binomial distribution.

The true frequencies of the genes in the pangenome are assumed to have a U-shaped Beta distribution (Lobkovsky *et al.* 2013, Colquhoun *et al.* 2021), where most genes are either core genes with a high true frequency or are much rarer accessory genes (Fig. 1B).

For a core gene G with unknown true frequency θ≥0.95, we estimate through simulations the probability of observing this gene X or fewer times over N genome samples (Fig. 1C). Due to missing sequence within assemblies, the distribution of observed gene frequencies is shifted to the left from the expected U-shape distribution, which is observed when analysing real MAG or SAG data (Supplementary Fig. S1). We then select the value of X such that the probability that a gene with true frequency θ≥0.95 is incorrectly identified as accessory, known as the false negative rate, is 5% (by default, but this value is tunable) or less: (observe G≤X times | θ≥0.95)≤5%.

The false negative rate is the probability of wrongly identifying a core gene as an accessory gene. There is a trade-off between the probability of missing true core genes (false negative rate) and the probability of wrongly identifying accessory genes as core genes (false positive rate). Lower false negative rates result in more true positives (correctly identified core genes) and more false positives (accessory genes incorrectly identified as core genes) (Supplementary Fig. S2). This effect is due to lower false negative rates resulting in lower adjusted core thresholds (Supplementary Fig. S3). Therefore, lower false negative rates prioritize correctly identifying core genes at the expense of incorrectly identifying accessory genes as core.

### 2.3 Simulated MAG dataset

*Escherichia coli* read datasets were randomly selected from (Kallonen *et al.* 2017) using SPAdes assemblies from (Page *et al.* 2016). Random sections of each genome were removed using a custom script (https://github.com/bacpop/CELEBRIMBOR/blob/main/simulate_pangenomes/remove_sequence.py), which removes a proportion of a genome assembly based on previously observed MAG completeness values from MGnify (Gurbich *et al.* 2023). Genome completeness estimates from CELEBRIMBOR for simulated data closely resembled those observed in real MAG data (Supplementary Fig. S4).

To quantify the effect of read coverage on core genome size prediction, simulated reads were also generated from the original assemblies using ART v2016.06.05 (Huang *et al.* 2012), simulating HiSeq 2500 paired-end reads with 5-, 10-, and 20-fold coverage. Reads were then assembled using metaSPAdes (Nurk *et al.* 2017) using parameters described in Page *et al.* (2016).

### 2.4 Pangenome analysis

Assemblies pre- and post-sequence removal were analysed using CELEBRIMBOR, using Bakta v1.8.2 (Schwengers *et al.* 2021) for gene prediction and annotation, MMseqs2 v14.7e284 (Steinegger and Söding 2017), or Panaroo v1.4.1 (Tonkin-Hill *et al.* 2020) in strict mode were used for clustering, and CheckM v1.2.2 (Parks *et al.* 2015) for genome completeness estimation. CELEBRIMBOR v0.1.0 and PPanGGOLiN v2.0.3 (Gautreau *et al.* 2020) were run on both pre- and post-sequence removal datasets. CELEBRIMBOR was run using core gene frequency thresholds in the range 70%–99%, with a rare gene frequency threshold and error rate set at 5% for all analyses. PPanGGOLiN was run with three pangenome partitions (‘-K 3’) on GFF files generated by CELEBRIMBOR via Bakta.

The number of genes labelled as ‘core’ and ‘rare’ by CELEBRIMBOR, or ‘persistent’ and ‘cloud’ by PPanGGOLiN were compared between pre- and post-sequence removal datasets across the range of core thresholds specified above. A full description of the simulation workflow used for CELEBRIMBOR benchmarking is available on Github (https://github.com/bacpop/MAG_pangenome_pipeline/tree/main/simulate_pangenomes). All analysis was performed on a workstation with 2 × 20 core Intel Xeon Gold CPUs.

## 3 Results

To simulate the effect of a dataset containing incomplete assemblies on core genome size estimation, we randomly removed sequence blocks from 500 full *E.coli* genome assemblies (Kallonen *et al.* 2017), with the amount of sequence removed from each assembly guided by previously observed MAG completeness values (Gurbich *et al.* 2023, Richardson *et al.* 2023). The dataset pre- and post-sequence removal was analysed using CELEBRIMBOR using two different methods of gene clustering: MMseqs2, a highly scalable protein sequence clustering method (Steinegger and Söding 2017), and Panaroo, a start-of-the-art pangenome analysis method which uses gene frequency and synteny to cluster and correct gene prediction errors (Tonkin-Hill *et al.* 2020).

As the core threshold is increased past 75%, the number of core genes identified falls dramatically, reaching zero at 90% without threshold adjustment for both MMseqs2 and Panaroo (Fig. 1D). Using CELEBRIMBOR greatly improves estimates of core genome size, staying close to the true total of core genes, independent of the clustering method. Furthermore, the number of accessory genes incorrectly predicted as core (false positives) is low, accounting for only 36/2850 (1.3%) core gene predictions when using a 95% core frequency threshold and 5% false negative rate using MMseqs2 for clustering (Supplementary Fig. S2).

Both clustering methods gave similar core genome size estimates after adjustment; at a core threshold of 95% and 5% false negative rate, MMseqs2 and Panaroo estimated a total of 3299 and 3434 core genes pre-sequence removal, and 2850 and 2936 using CELEBRIMBOR post-sequence removal, whilst only 2 and 3 core genes were identified without adjustment respectively (Supplementary Table S1). The slightly larger core genome estimates provided by Panaroo over MMseqs2 are indicative of the more accurate orthologue detection strategies used by Panaroo, including the use of iterative similarity thresholds and synteny during clustering. Altering Panaroo’s stringency settings did not affect estimates of core genome size, although more stringent removal of low-frequency genes reduced the number of estimated rare genes (Supplementary Fig. S5). In comparison, PPanGGOLiN estimated a total of 3711 and 4099 core genes pre- and post-sequence removal, respectively. A higher-core genome size estimate post-sequence removal is indicative of incorrect assignment of accessory genes as core, meaning PPanGGOLiN’s method for threshold adjustment is overly sensitive and results in false positives. CELEBRIMBOR is by default more conservative, resulting in more false negatives, although this trade-off can be reduced by decreasing the false negative rate.

MAG and SAG incompleteness can occur due to systematically low read coverage, resulting in genome regions being incorrectly assembled or not assembled at all (Chen *et al.* 2020). As CELEBRIMBOR assumes all genes in a pangenome have an equal chance of being missed, systematically low coverage detrimentally affects core genome size estimation, resulting in notably lower accuracy with low read-coverage assemblies (Supplementary Fig. S6).

To compare computational efficiency, a series of datasets up to 1500 simulated MAGs in size from (Kallonen *et al.* 2017) were analysed using CELEBRIMBOR running MMseqs2 or Panaroo. Using MMseqs2 gave consistently lower runtimes compared to Panaroo, although runtime for both workflows scaled linearly with dataset size (Supplementary Fig. S7). Maximum memory usage can be adjusted by the user via the command line interface of Snakemake, and so was not measured here. Overall, MMseqs2 provides greater computational scalability in clustering over Panaroo, which is of particular importance when including increasingly large numbers of genomes in analyses.

## 4 Discussion

MAGs and SAGs are a crucial resource in the analysis of bacterial pangenome diversity, particularly for species that cannot be cultured under artificial conditions. CELEBRIMBOR enables a parametric recapitulation of the core genome using MAGs and SAGs, which would otherwise be unidentifiable due to missing sequences resulting from errors in the assembly and binning processes. CELEBRIMBOR implements both computationally efficient and accurate clustering workflows; MMseqs2, which scales to millions of gene sequences (Steinegger and Söding 2017), and Panaroo, which uses sophisticated network-based approaches to correct errors in gene prediction and clustering (Tonkin-Hill *et al.* 2020).

We show that CELEBRIMBOR can accurately estimate the size of the core genome in simulated MAG data, far outperforming methods where no threshold adjustment is made. Panaroo and MMseqs2 were not able to identify any core genes past the 90% threshold, despite Panaroo including a method for ‘re-finding’, which identifies missing gene predictions resulting from mutations or assembly errors (Tonkin-Hill *et al.* 2020). However, the integration of Panaroo into CELEBRIMBOR combines Panaroo’s error correction methods with CELEBRIMBOR’s core threshold adjustment, resulting in highly accurate quantification of gene frequencies of both core and lower frequency genes.

A crucial assumption underlying core genome threshold adjustment models (including in this work) is that genes in the genomes of MAGs and SAGs are assumed to be missing completely at random. However, if certain parts of the genome are more liable to errors—because of uneven read coverage, repetitive genes, or genes highly conserved between species—then such data would violate the assumption of regions missing completely at random. A consequence would then be that true core genes that are not missing at random fail to be labelled as core genes.

CELEBRIMBOR is a rapid pangenome analysis and core threshold adjustment pipeline designed for the analysis of large MAG and SAG datasets. It enables researchers working exclusively with MAGs and SAGs to accurately identify core genes, facilitating epidemiological and evolutionary analysis even in the presence of missing data, as well as investigation of sequencing and assembly issues that lead to gene drop out in gene presence/absence matrices.

## Supplementary Material

btae542_Supplementary_Data

## Data Availability

Code for CELEBRIMBOR and pangenome simulations is available on Github (https://github.com/bacpop/CELEBRIMBOR). Code for cgt is also available on Github (https://github.com/bacpop/cgt).
