# Supplementary material for: CELEBRIMBOR: core and accessory genes from metagenomes
Source: Bioinformatics. 2024 Sep 19;40(9):btae542. doi: 10.1093/bioinformatics/btae542 (PMC11422503; doi:10.1093/bioinformatics/btae542)
Supplement: btae542_Supplementary_Data [file btae542_supplementary_data.docx]

## Supplementary Figures


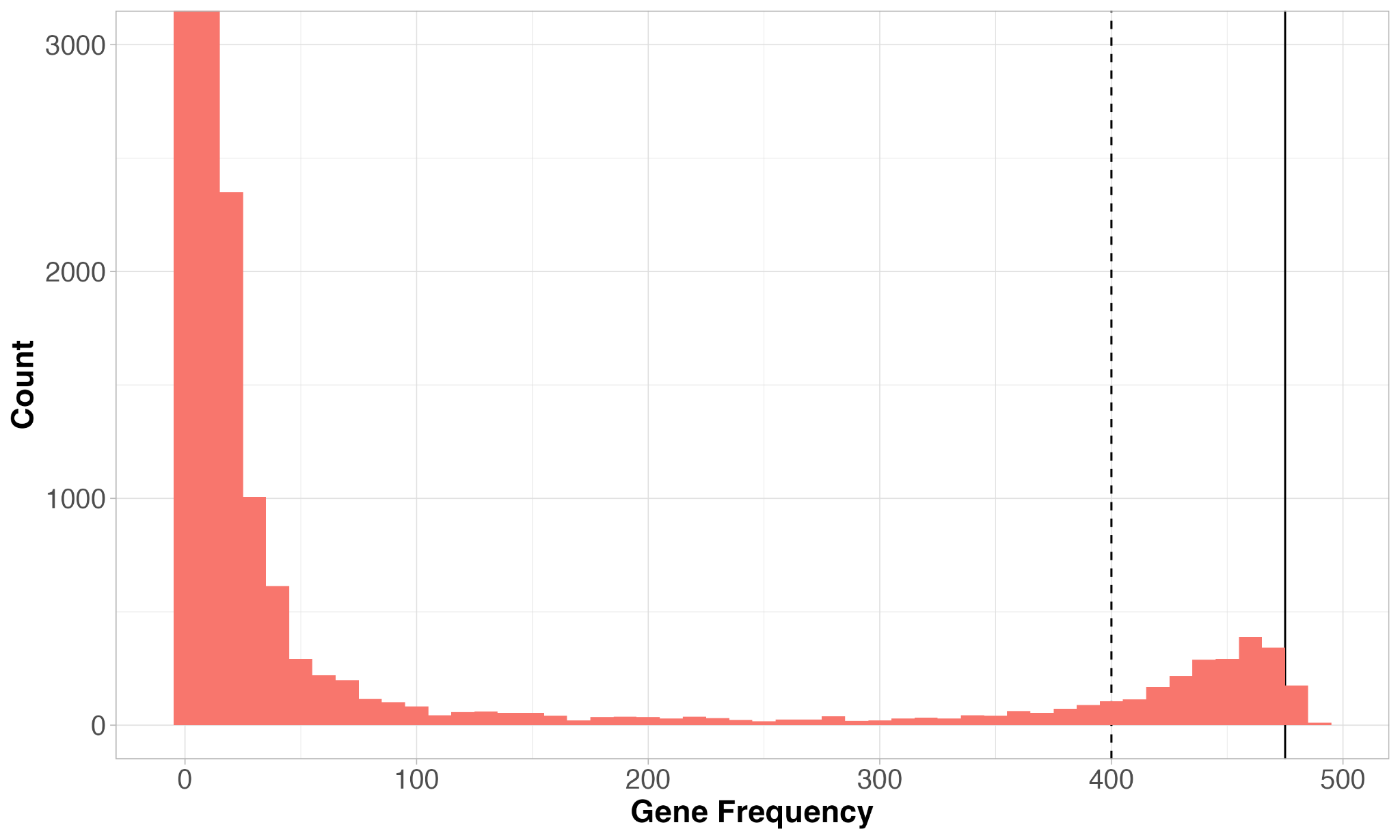


Supplementary Figure 1: Gene frequency distribution of 500 Bacteroides uniformis MAGs from the MGnify database ([Gurbich et al., 2023)](https://paperpile.com/c/nnSJ5t/xM7f+ZRaz). The solid horizontal line represents the 95% core threshold (No. genes in cluster = 475); the dashed line represents the adjusted core threshold (80%) predicted by CELEBRIMBOR (No. genes in cluster = 400). MMseqs2 was used for clustering. Note: Y-axis cut at Count=3000 for ease of visualisation due to a large number of low frequency genes.


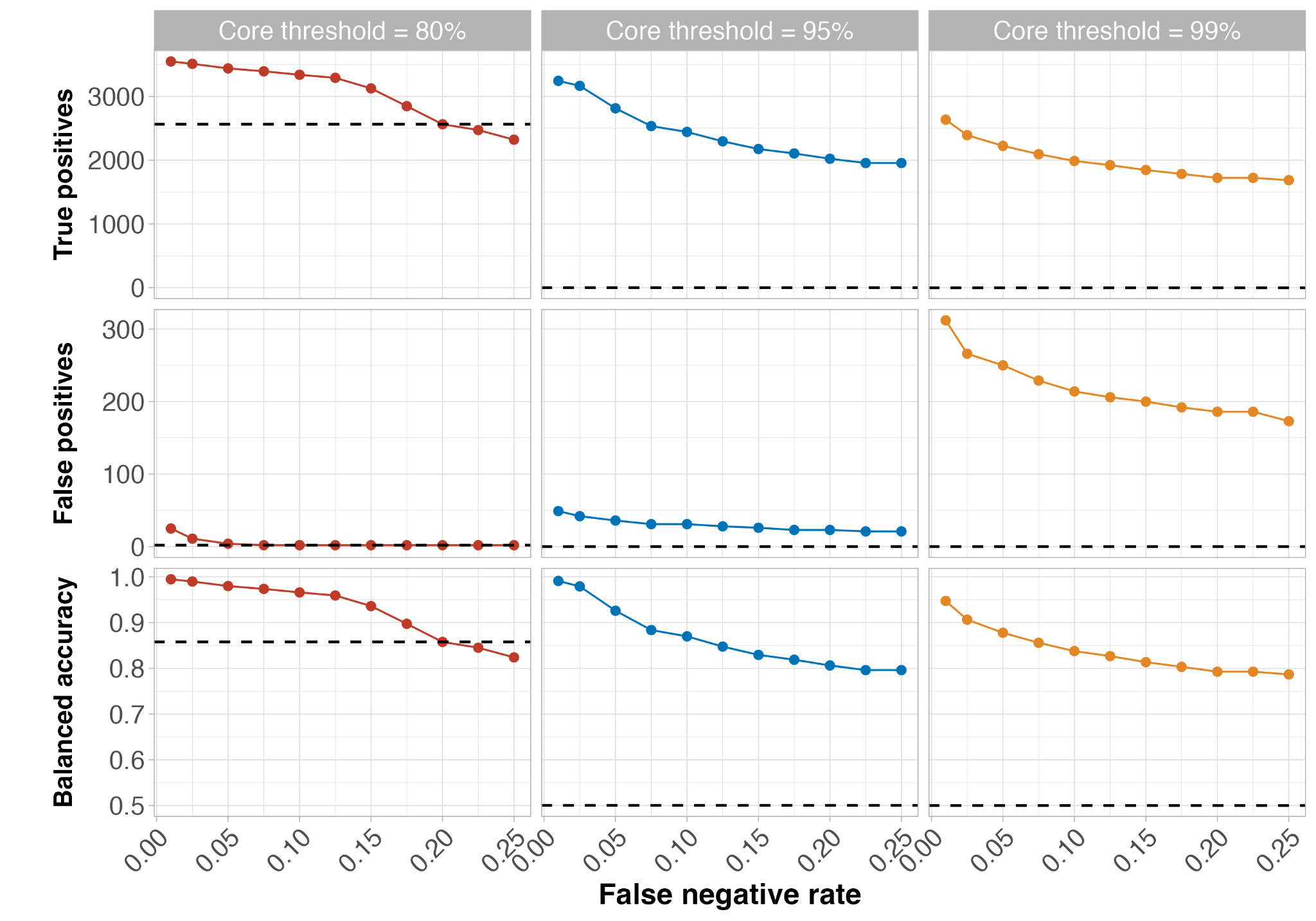


Supplementary Figure 2: The effect that varying the false negative rate has on numbers of true positives, false positives and balanced accuracy. True positives refer to true core genes (≥X% frequency prior to sequence removal) which were correctly identified as core genes by CELEBRIMBOR. False positives refer to accessory genes (<X% frequency prior to sequence removal) which were incorrectly identified as core genes by CELEBRIMBOR. Columns refer to the pre-specified core frequency threshold. Balanced accuracy is calculated as (Sensitivity + Specificity) / 2, where Sensitivity = TP / (TP + FN) and Specificity = TN / (TN + FP). TP = True Positive, FN = False Negative, TN = True Negative, FP = False Positive. MMseqs2 was used for clustering. Black dotted lines refer to the number of core genes identified without CELEBRIMBOR adjustment.


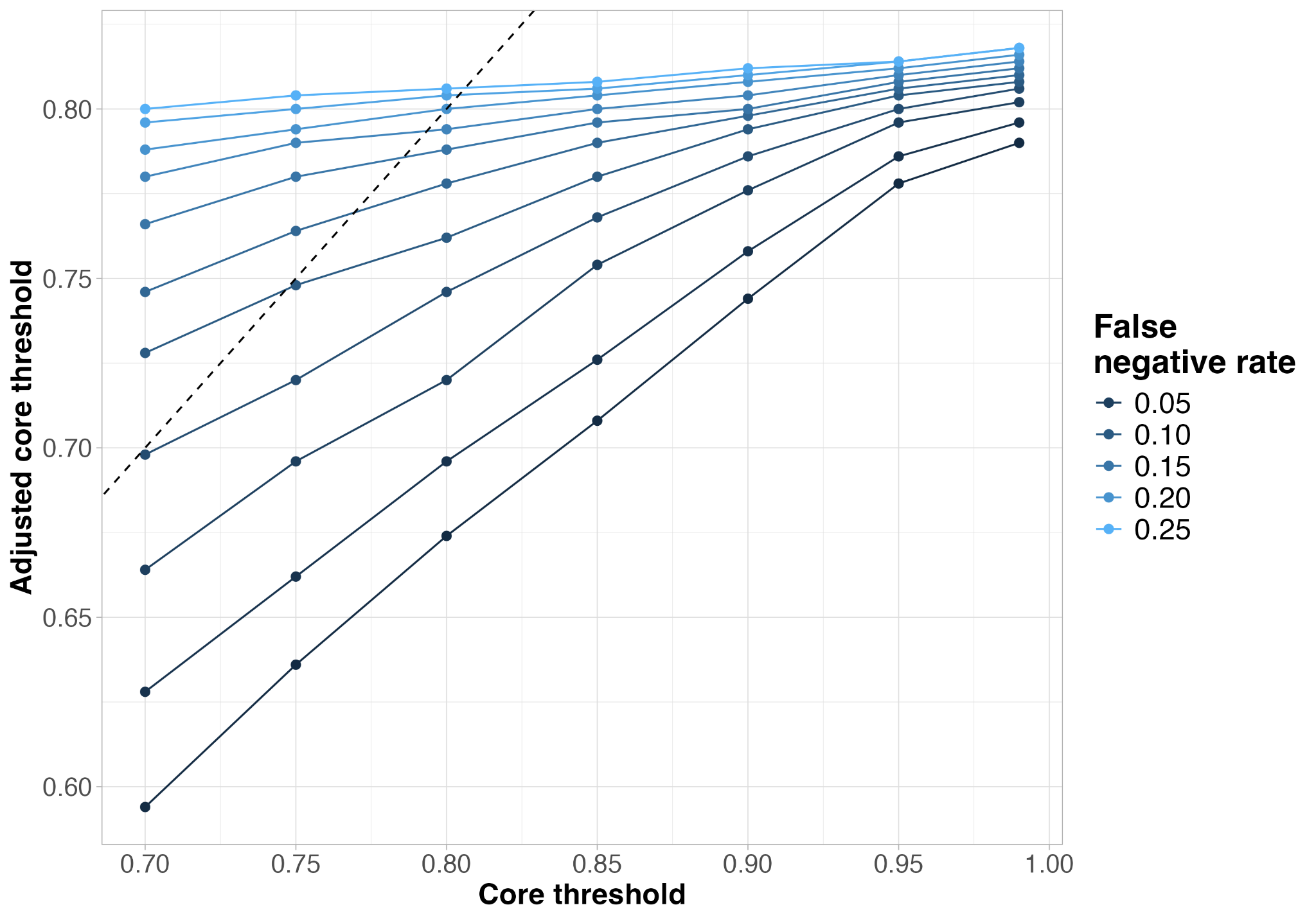


Supplementary Figure 3: The effect that varying the false negative rate has on the relationship between the original core and adjusted core threshold. MMseqs2 was used for clustering. Black dotted line indicates y=x.


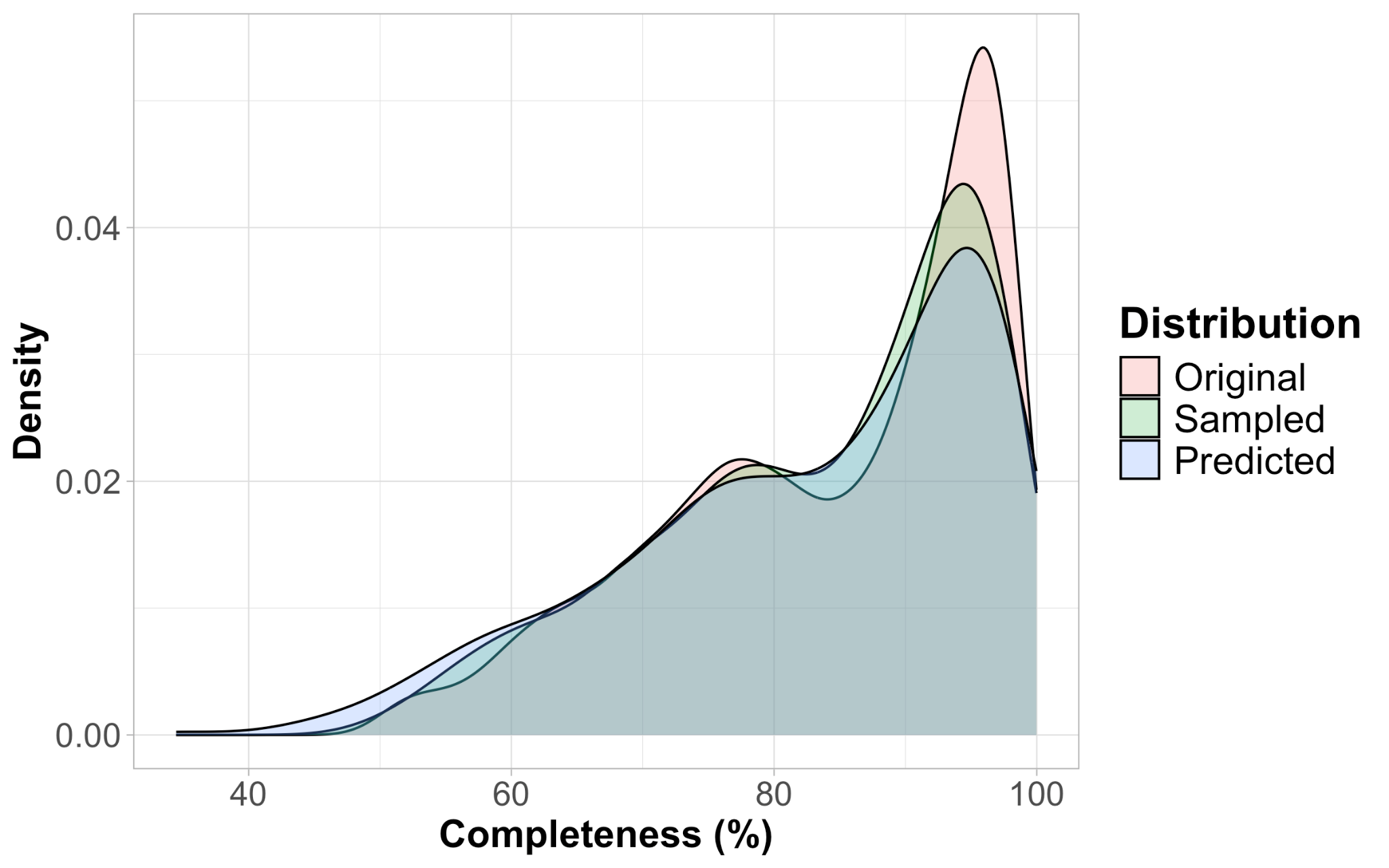


Supplementary Figure 4: Completeness distributions for simulated assemblies. ‘Original’ refers to the distribution retrieved from MGnify ([Gurbich *et al.*, 2023)](https://paperpile.com/c/nnSJ5t/xM7f+ZRaz). ‘Sampled’ refers to values used for simulated assemblies. ‘Predicted’ refers to values inferred by CheckM1 on simulated assemblies.


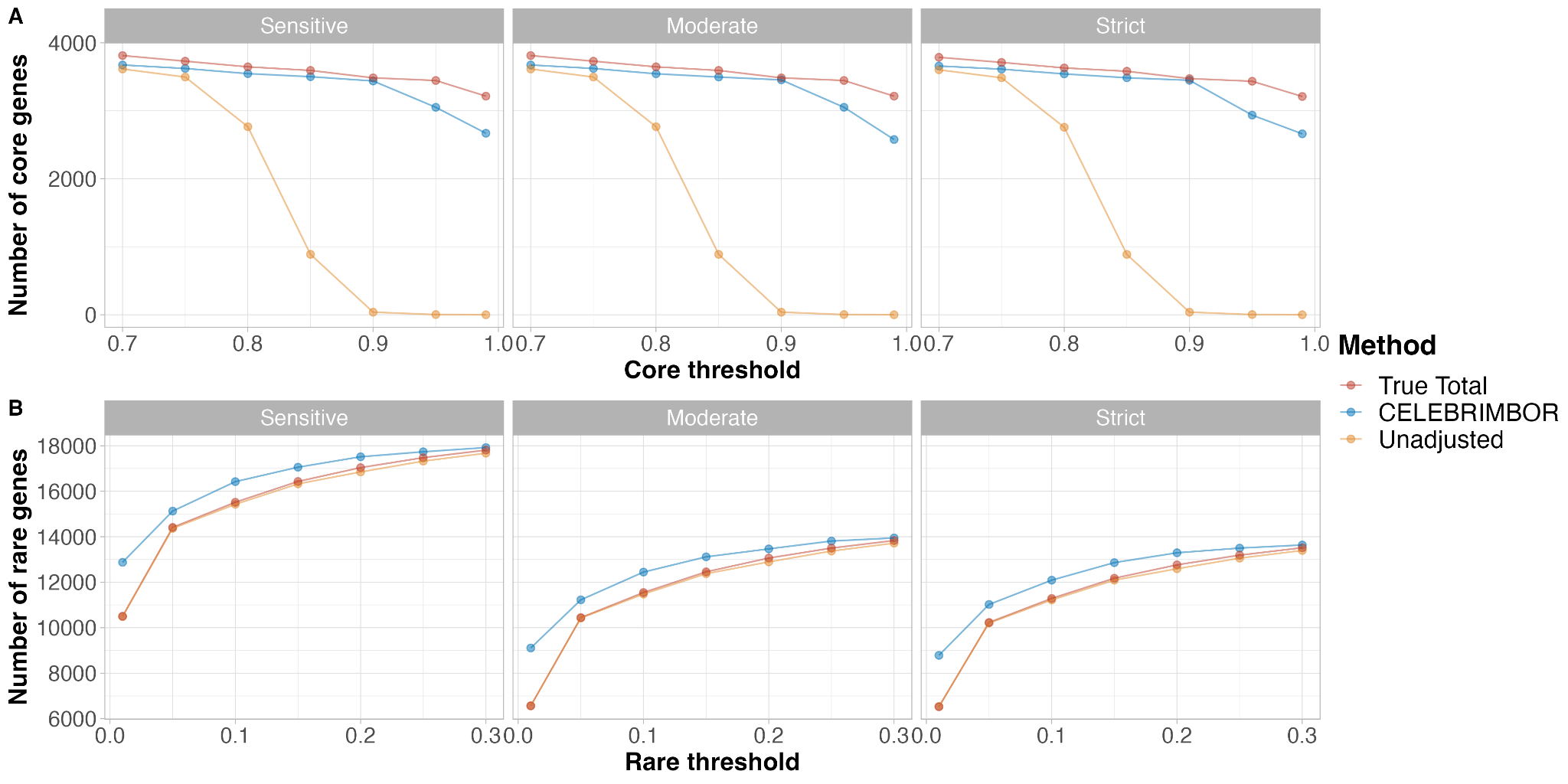


Supplementary Figure 5: Comparison of Panaroo stringency settings on the estimated number of (**A**) core and (**B**) rare genes using CELEBRIMBOR. For (**A**), the rare threshold was set at 5%; for (**B**) the core threshold was set at 95%. For both (**A**) and (**B**), the error threshold was set at 5%. Columns describe Panaroo stringency settings; ‘Sensitive’, ‘Moderate’ and ‘Strict’ (see [(Tonkin-Hill *et al.*, 2020)](https://paperpile.com/c/nnSJ5t/SEAx) for description of settings).


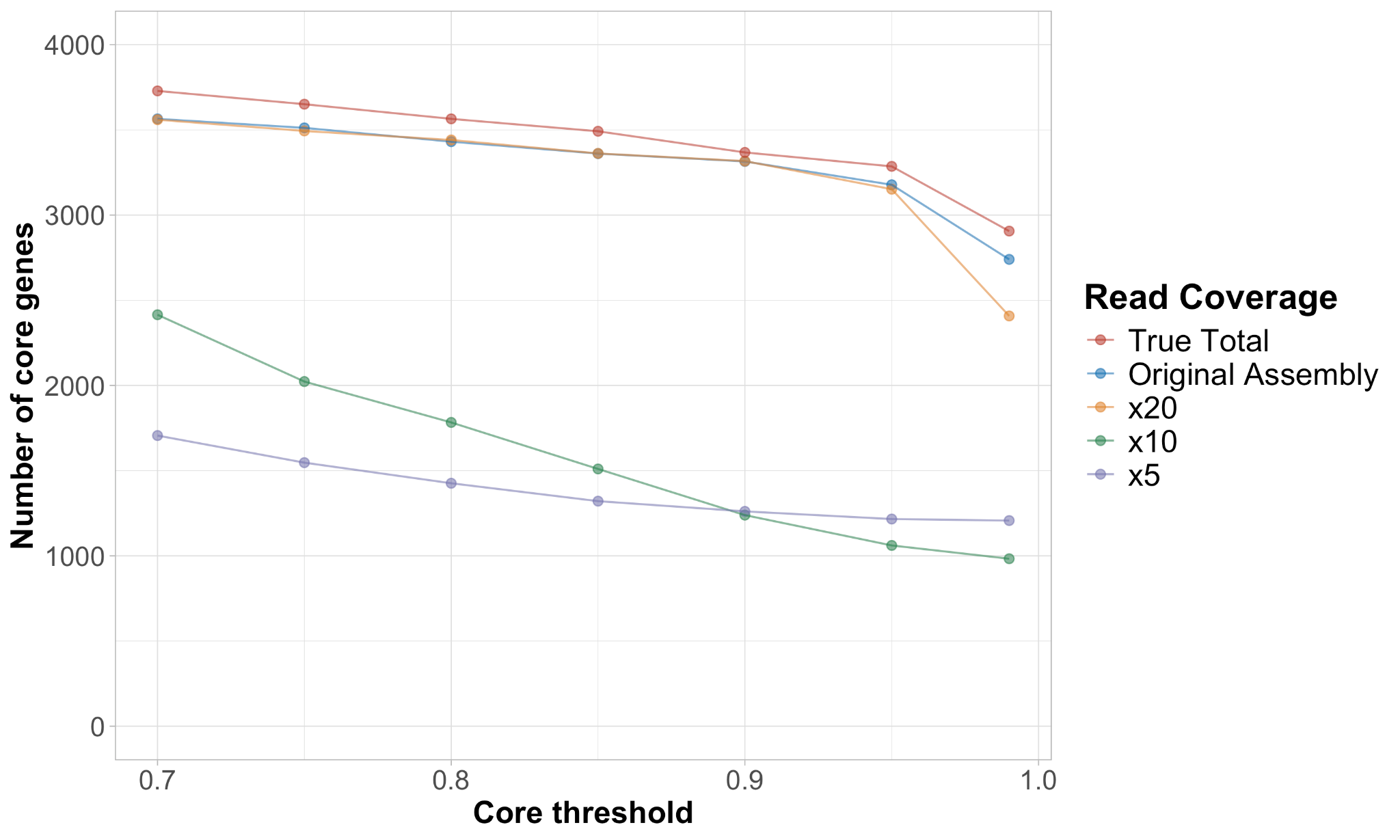


Supplementary Figure 6: Effect of assembly read coverage on core genome size estimates. Core genome size estimation was conducted on the same 500 *E. coli* assemblies analysed in Figure 1 using MMseqs2 for clustering. ‘True Total’ refers to the number of core genome size estimates from original assemblies based on an unadjusted core frequency threshold of 95% frequency. ‘Original Assembly’ refers to core genome size estimates by CELEBRIMBOR on the original assemblies. ‘xN’ refers to the mean fold coverage of the assemblies, where N refers to the average number of read bases aligning to the locus in the assembly, with core genome estimates generated by CELEBRIMBOR.


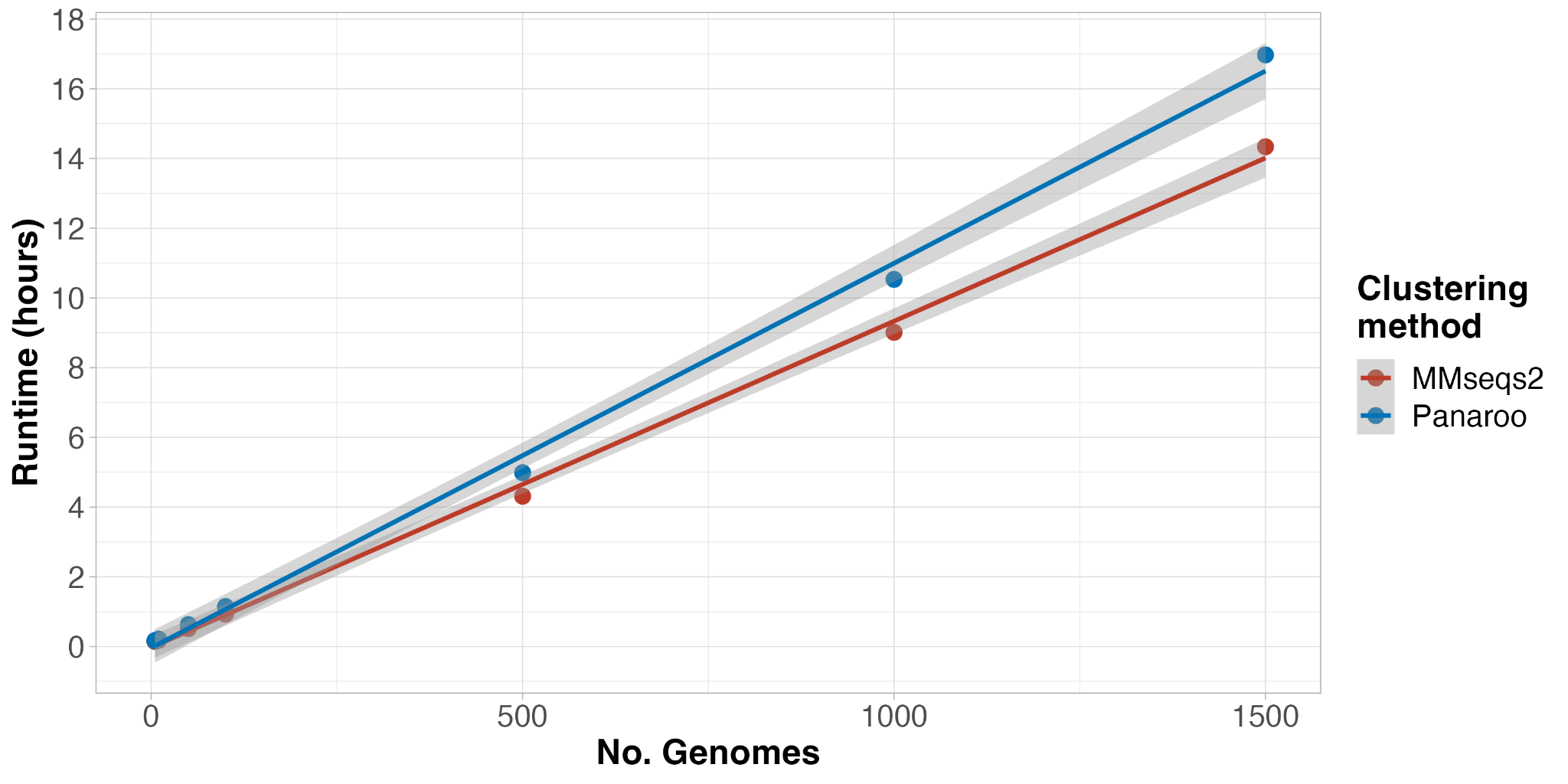


Supplementary Figure 7: Runtime comparison of clustering methods used in CELEBRIMBOR. Grey boundaries indicate 95% confidence intervals for linear regression. Comparisons were conducted on simulated MAGs from *E. coli* genomes from [(Kallonen *et al.*, 2017)](https://paperpile.com/c/nnSJ5t/kKXL). CELEBRIMBOR was run with 16 threads for all analyses.

## Supplementary Tables

Supplementary Table 1: Number of genes assigned to frequency compartments by different clustering algorithms. For clustering using MMseqs2 and Panaroo, CELEBRIMBOR was run with core and rare thresholds set to 95% and 5%, respectively, and error threshold at 5%. Intermediate genes are found at frequency 5% > X > 95%.

| **Clustering method** | **Adjustment** | **No. core** | **No. intermediate** | **No. rare** |
| --- | --- | --- | --- | --- |
| MMseqs2 | True | 3299 | 4929 | 24726 |
|  | CELEBRIMBOR | 2850 | 3973 | 26131 |
|  | Unadjusted | 2 | 7791 | 25161 |
| Panaroo | True | 3434 | 4646 | 10232 |
|  | CELEBRIMBOR | 2936 | 3989 | 11022 |
|  | Unadjusted | 3 | 7739 | 10205 |
| PPanGGOLiN | True | 3711 | 2955 | 15149 |
|  | Adjusted | 4099 | 2824 | 14453 |
